# Layer-by-Layer-Assembled Polyaniline/MXene Thin Film and Device for Improved Electrochromic and Energy Storage Capabilities

**DOI:** 10.1021/acsapm.4c01774

**Published:** 2024-10-09

**Authors:** Dejuan Lu, Jian Li, Dashui Zhang, Lina Li, Zhangfa Tong, Hongbing Ji, Junxin Wang, Caixia Chi, Hui-Ying Qu

**Affiliations:** † Guangxi Key Laboratory of Petrochemical Resource Processing and Process Intensification Technology, School of Chemistry and Chemical Engineering, Guangxi University, Nanning 530004, China; ‡ Department of Materials Science and Metallurgy, University of Cambridge, 27 Charles Babbage Road, Cambridge CB3 0FS, United Kingdom; § Heilongjiang Province Key Laboratory of Environmental Catalysis and Energy Storage Materials, Food and Pharmaceutical Engineering College, Suihua University, Suihua 152061, China

**Keywords:** electrochromic, energy storage, polyaniline, MXene, device

## Abstract

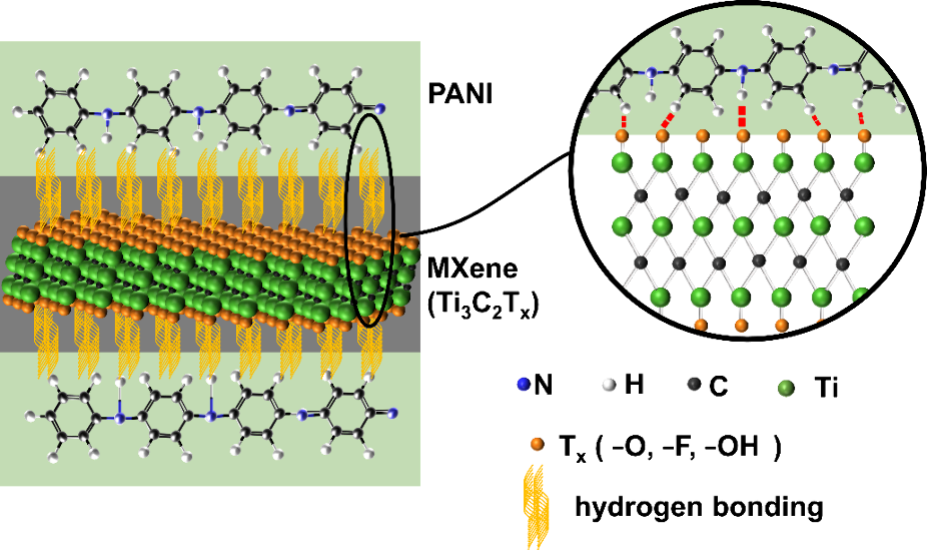

Polyaniline (PANI) is an attractive electrochromic and storage material due to its reversible and sustainable electrochemical redox processes. However, the insufficient surface area and excessive charge intercalation after long-term cycling results in limited charge capacitance and unsatisfactory cycling stability. In this work, we demonstrate an innovative method to increase PANI’s electrochromic and energy storage performance by incorporating MXene, to enhance electrochemical activity and reveal more active areas of ion/electron intercalation/deintercalation and charge transfer. The hydrogen bonds formed between N–H, C–H, and C–N of PANI and −OH and −O surface functional terminations of MXene further enhance the interface interaction. With substantial optical transmittance modulation and charge capacitance, excellent coloration efficiency, and outstanding durability, the PANI/MXene thin film demonstrates exceptional color-switching and energy storage characteristics. Furthermore, the sandwich device with a PANI/MXene thin film as the positive electrode and zinc foil as the negative electrode demonstrates exceptional electrochromic and Zn^2+^ storage capability. This work raises possibilities for next-generation intelligent energy conversion and storage technologies and offers fresh perspectives on the design of ionic devices.

## 1 Introduction

Exploring efficient pathways for storage technologies and energy conversion, including batteries and supercapacitors, helps address the energy crisis and environmental pollution issues. Combining their energy storage performance with other special functions such as electrochromism, a phenomenon in which an applied electric field causes a material’s optical characteristics to undergo a reversible color transformation, can achieve versatile applications.^1−5^ For example, the color of the energy storage device’s appearance can be used to determine if it is charging, or an electrochromic smart window can be self-powered to regulate indoor lighting.^6−9^ Various materials, including inorganic WO_3_,^10^ NiO,^11^ TiO_2_,^12^ Co_3_O_4_,^13^ V_2_O_5_,^14^ and organic polypyrrole,^15^ polyaniline (PANI),^16^ are promising candidates for electrochromic energy storage devices. During the charging/discharging process, light transmission of these materials can be modulated sustainably and reversibly according to the amount of charge.

PANI has garnered the most interest among the above-mentioned materials because of its multicolor, fast response, remarkable coloring efficiency, and efficient energy storage performance.^17−20^ However, during the electrochemical redox processes of PANI, dopant ions originated from the electrolyte are constantly intercalated and deintercalated between the PANI chains. Excessive intercalation of charge due to the trapped ions after long-term cycling results in reduced electrochemical activity and destroyed chain skeleton of PANI, thus leading to unsatisfactory cycling stability.^21−23^ In addition, the insufficient surface area and active sites also limit the number of ions/electrons involved during the electrochromic and energy storage process of PANI.^24^ Compounding PANI with other nanomaterials is one of the most efficient strategies to raise PANI’s cycling stability and specific capacity.^25,26^ For instance, Nguyen et al. created a PANI-WO_3_ electrochromic film. Compared to the pure PANI film, the greater specific surface area of PANI-WO_3_ film facilitates the intercalation/deintercalation of H^+^ and improves the cycle durability.^27^ Because Tran et al. constructed the MnO_2_–PANI hybrid, the high theoretical capacity of MnO_2_ allowed for achieving a high areal capacity of 0.127 mAh·cm^–2^ of 0.072 mA·cm^–2^.^28^

A two-dimensional transition metal carbide crystal having −OH, −O, and −F surface functional terminations is designated as Ti_3_C_2_T_*x*_ MXene.^29,30^ It is produced by carefully etching the Al atomic layer of the parent Ti_3_AlC_2_ phase, the alternating Ti and C layers as well as the highly ordered arrangement endows Ti_3_C_2_T_*x*_ MXene with a sizable specific surface area and strong electrical conductivity for transferring charge to expose more active sites, serving as a promising candidate for energy storage.^31^ Razal et al. have achieved a conductivity of ∼15100 S·cm^–1^ of MXene flakes measuring 214 nm thick.^32^ The specific surface area of the porous MXene prepared by Zhang et al. can reach up to 85 m^2^·g^–1^.^33^ In addition, Ti_3_C_2_T_*x*_ MXene can enhance chemical and hydrogen bondings between materials.^34,35^ Therefore, by introducing MXene, the preparation of MXene/PANI-based composites can solve the problems of PANI’s cycling stability and specific capacity. For example, Wu et al. demonstrated that amino-Ti_3_C_2_/polyaniline (N-Ti_3_C_2_/PANI) composites were used as supercapacitor electrodes with a capacitance retention rate of nearly 85% after 1000 charge/discharge cycles.^36^ Xie et al. reported PANI/g-C_3_N_4_/MXene composites were used as electrodes for supercapacitors and showed excellent cycling stability with a retention rate as high as 91.1% after 1000 charge/discharge cycles.^37−39^ Salles et al. prepared Ti_3_C_2_T_*x*_ MXene as an electrochromic device with a transmittance of 12%.^40^ However, few studies have addressed electrochemical properties of PANI/MXene composites on the dual function of electrochromism and energy storage.

Herein, we demonstrate an effective strategy to improve PANI’s durability and energy storage performance by assembling the MXene layer into the PANI film using the layer-by-layer (LBL) technique. MXene is constructed as an intermediate layer in the middle of ductile PANI to avoid the direct exposure of MXene to air, which hinders the contact between MXene and molecular oxygen and avoids the restacking/aggregation and susceptibility to oxidization of MXene. Hydrogen bonds formed between PANI and surface functional terminations of MXene enhance the interface interaction. The excellent conductivity of MXene facilitates ion/electron intercalation/deintercalation and charge transfer during the electrochromic and charging/discharging processes. As a positively charged anodic electrochromic material with reversible redox properties, PANI is attracted to the negatively charged MXene, which prevents the 2D nanosheets from restacking. The platform formed by their mutual attraction provides additional channel space for the storage and transport of electrolyte ions, which improves the reversibility of cycling and avoids excessive charge intercalation during long-term electrochemical cycling. The inserted MXene also contributes to decreasing the PANI layer’s thickness, expanding the area’s surface, and increasing the active sites so that more ions/electrons can be involved in PANI’s electrochromic and energy storage process. A sandwich device featuring a positive electrode made of the PANI/MXene film and a negative electrode made of zinc foil was also constructed. It demonstrated exceptional performance in electrochromic and Zn^2+^-storage, with huge areal capacitance and optical modulation, excellent coloration efficiency, and remarkable optical and electrochemical durability.

## 2 Experimental Section

### 2.1 Materials

Aniline (≥99.5%) and poly(vinyl alcohol) (PVA, 88.0 mol %) were acquired from Macklin Biochemical Co., Ltd. *p*-Toluenesulfonic acid (TsOH, ≥99.0%) was provided by Sinopharm Chemical Reagent Co., Ltd. Guangdong Guanghua Sci-Tech Co., Ltd., provided ammonia (NH_3_, 25–28%), formic acid (HCOOH, ≥88.0%), and ammonium persulfate (APS, ≥98.0%). Chengdu Chron Chemicals Co., Ltd., provided anhydrous ethanol (C_2_H_5_OH, >99.7%) and hydrochloric acid (HCI, 36 wt %). Foshan Xinxi Technology Co., Ltd., provided the MXene (10 mg·mL^–1^, Ti_3_C_2_T_*x*_, ≥99.9%) aqueous dispersion. Zinc foils (99.9% zinc) were provided by Tianjin Shentai Chemical Reagent Co. Foshan Yuanjingmei Glass Co. provided indium–tin oxide (ITO)-coated glass substrates with a resistance of 6 Ω·sq^–1^. Deionized (DI) water was made in our laboratory. No additional purification was applied to any of the compounds.

### 2.2 Preparation of PANI

PANI was created using a standard chemical oxidative polymerization process regarding the literature.^41^ First, 4.66 g of aniline was dissolved in 25 mL of a 1 mol·L^–1^ solution of HCl/TsOH. After concentrated hydrochloric acid was added to adjust the pH to 1.0, the mixture was labeled A. A total of 14.26 g of APS was dissolved in 25 mL of DI water, and the resulting solution was labeled B. A and B were combined, allowed to cool to 0 °C for 3 h, and then filtered and rinsed with DI water. To obtain the dedoped PANI, the substance was dissolved in 100 mL of ammonia solution and agitated for 24 h. It was then filtered and cleaned with ethanol and DI water, consecutively. At last, a dark-blue PANI powder was obtained using a 12-h vacuum-drying process at 80 °C.

### 2.3 Preparation of the PANI/MXene, PANI, and MXene Thin Films

ITO-coated glass substrates (1 × 2 cm^2^) were ultrasonically cleaned in three steps using acetone, ethanol, and DI water and then dried at 120 °C on a heating plate. After DI water was added to dilute the MXene aqueous dispersion to 5 mg·mL^–1^, it was sonicated for 10 min.

The PANI/MXene thin film was prepared as follows. To make the PANI solution, 0.1 g of PANI was first dissolved in 25 mL of formic acid while being stirring for 12 h. A total of 50 μL of PANI solution was sprayed onto the cleaned ITO-coated glass substrates four times to obtain the PANI layer. After that, 30 μL of 5 mg·mL^–1^ MXene dispersion was spin-coated onto the PANI layer at 700, 1000, and 3000 rpm for 30 s each. Last, to create the PANI/MXene composite thin film, the PANI layer preparation steps were repeated. Also, thin films from PANI and MXene were prepared for comparison. The PANI solution’s spraying process was repeated eight times for the PANI thin film.

### 2.4 Construction of the Electrochromic Energy Storage Apparatus

First, 10 g of PVA and 3.4 g of ZnCl were dissolved in 100 mL of DI water. The pH of the solution was adjusted to 2.5 by adding HCl/TsOH, stirred at 90 °C until clear, then cooled to room temperature, and used as the gel electrolyte. Next, a zinc foil was positioned between the glass substrate and the PANI/MXene thin film, and the edges of both were taped with 3 M double-sided tape, which had a thickness of 1.0 mm. Ultimately, the gel electrolyte was injected to create an electrochromic energy storage device, where the positive electrode was the PANI/MXene thin film and the negative electrode was zinc foil.

### 2.5 Characterization

X-ray diffraction (XRD, Rigaku Ultima IV) measured the crystalline structure of thin films. Surface morphology and thickness were assessed via scanning electron microscopy (SEM, TESCAN MIRA LMS; 15 kV acceleration voltage). The chemical structure was examined by Raman (WiTech alpha300R) with a 532 nm wavelength laser, Fourier transform infrared (FTIR, Thermo Scientific Nicolet iS20), and attenuated-total-reflectance Fourier transform infrared (ATR-FTIR, Thermo Scientific Nicolet iS20). A BET analyzer (ASAP 2460) was used to measure the specific surface area, and the pore volume was determined via the BJH analytical technique. X-ray photoelectron spectroscopy (XPS, Thermo Scientific K-Alpha) was transported at a voltage of 12 kV (1486.6 eV) using monochromatic Al Kα irradiation, number of scans of 1, a step size of 1 eV, and a total acquisition time of 2 min 16.1 s, and the characteristic binding energy of C 1s at 284.8 eV was used for calibration.

### 2.6 Electrochemical and Electrochromic Measurements

An electrochemical workstation (Shanghai Chenhua Instrument Co., Ltd., CHI760E) with a three-electrode setup had been utilized to conduct electrochemical measurements, including cyclic voltammetry (CV), chronoamperometry (CA), galvanostatic charge/discharge (GCD), and electrochemical impedance spectroscopy (EIS) of thin films. The accuracy of potentials and currents applied by our electrochemical workstations is ±0.1%. The working electrodes of the workstation were PANI, MXene, and PANI/MXene thin films; the reference electrode proved Ag/AgCl; the counter electrode experienced platinum foil; the electrolyte proved 0.01 M HCl/TsOH aqueous solution. EIS measurements were conducted over a frequency range of 0.01 Hz to 1000 kHz. The device with zinc foil as the negative electrode and PANI/MXene thin film as the positive electrode was measured for CV and GCD under a two-electrode system. In situ optical transmittance of thin films and the device in the 350–1000 nm wavelength range were investigated in conjunction with the electrochemical data. A vessel with only the electrolyte was utilized to achieve the 100% level for the films’ transmittance. In contrast, air was utilized to achieve the same level for the device’s transmittance. More than five samples of each thin film were used for each measurement.

The coloration efficiency was computed as^42^
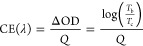
1where *Q* represents the intercalated charge, ΔOD represents the optical density, and *T*_c_ and *T*_b_ are the transmittance of thin films in their colored and bleached states, respectively, at a specified wavelength.

The areal capacitance was computed from the GCD data by eq 2:^12^
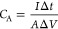
2where the discharge voltage range is represented by Δ*V* (V), the working electrode area is *A* (cm^2^), the discharge time is Δ*t* (s), and the discharge current is *I* (mA).

The charge storage behavior was analyzed using the power-law rule:^43^
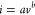
3
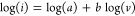
4where *a* and *b* are empirical parameters and *b* is calculated using a log(*i*)–log(*v*) plot.

The following formulas were used to quantify the contributions of diffusion and capacitive-controlled charge storage:^44^
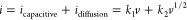
5
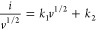
6where *i* is the current density, *v* is the scan rate, and *k*_1_ and *k*_2_ are constants.

The device’s energy and power density were computed using^45^
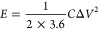
7
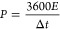
8where the discharge voltage range is Δ*V* (V), the discharge time is Δ*t* (s), and the device’s area capacity is *C* (mF·cm^–2^).

## 3 Results and Discussion

**Figure 1:**
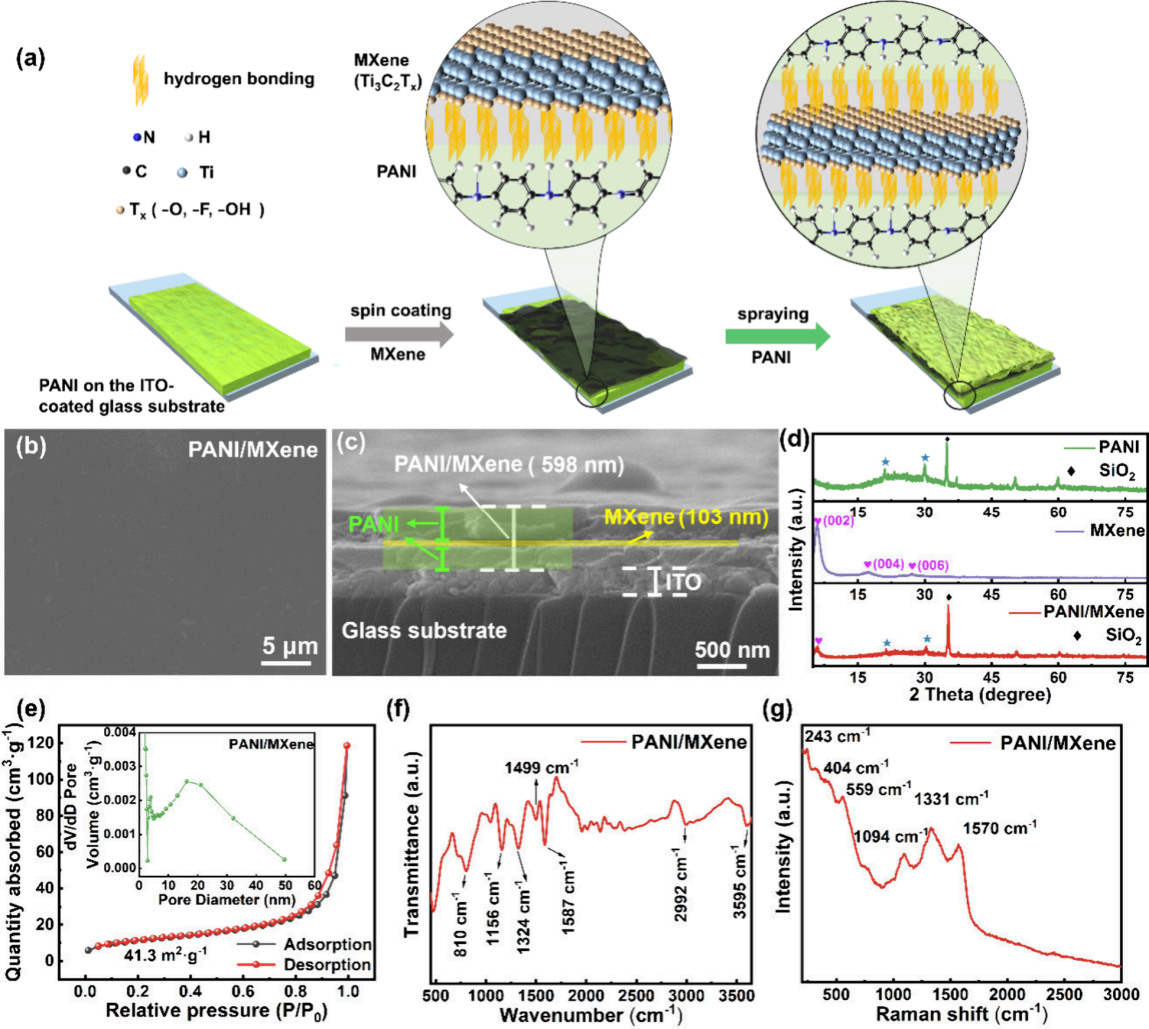
Synthesis and characterization of the PANI/MXene thin film. (a) Synthesis scheme. (b) Surface SEM image. (c) Cross-sectional SEM image. (d) XRD spectra of the PANI thin film, MXene powder, and the PANI/MXene thin film on the glass substrate. (e) N_2_ adsorption/desorption isotherms of the PANI/MXene powder (the inset represents the pore size distribution). (f) ATR-FTIR spectrum. (g) Raman spectrum.

Figure 1a shows the schematic diagram of the PANI/MXene thin film preparation process, as described in the experimental section. The PANI powder produced through chemical oxidative polymerization is in the shape of particles (Figure S1). PANI’s dissolution and then spraying will form a dense thin film with no obvious structure (Figure S2a). The dense morphology increases the difficulty of H^+^ in the embedding and dislodging process.^46^ Dense and homogeneous PANI was coated on the PANI/MXene thin film’s surface (Figure 1b). The 103-nm-thick MXene nanosheet layer was firmly stacked in the middle of the PANI layer on both sides of PANI/MXene with a total thickness of 598 nm, as displayed in the cross-sectional SEM (Figure 1c). For comparison, the morphology of the PANI and MXene thin film is shown in Figure S2a–c.

The XRD patterns of PANI, MXene, and PANI/MXene are displayed in Figure 1d. The PANI/MXene thin film exhibits diffraction peaks at 21.0° and 30.0° originating from PANI and a significantly reduced peak at 5.94° from the sandwiched MXene. The original MXene membrane in the dry state had a (002) peak located near 2θ = 5.94°, with a *d* spacing of 14.88 Å calculated from Bragg’s equation. In the composite thin films, the (002) peak located near 2θ = 5.76°, with a *d* spacing of 15.34 Å. The membrane increased by 0.46 Å. The hydrogen bonding between PANI and MXene increases the spacing of MXene, which acts as an intercalating agent to increase the layer spacing of the composite thin film, effectively providing more active sites, fast mass transfer, and sufficient space for electrolyte deposition/decomposition, thus improving the ion storage capacity of supercapacitors.^47^ The specific surface area and pore size distribution were investigated using the N_2_ adsorption isotherm (Figures 1e and S2d,e). The average pore size of the PANI/MXene thin film is 19.8 nm, smaller than 28.0 nm of the PANI thin film, leading to a larger BET surface area of 41.3 m^2^·g^–1^ than the PANI thin film for 33.8 m^2^·g^–1^; the increased specific surface area of the PANI/MXene thin film facilitates ion/electron intercalation/deintercalation and transfer during the electrochromic and charging/discharging processes.

Additional techniques for examining the thin films’ composition and structural properties included FTIR, ATR-FTIR, and Raman spectroscopy. The distinctive peaks of PANI (Figure S2f) and MXene (Figure S2g) are seen in the PANI/MXene thin film’s ATR-FTIR spectrum (Figure 1f). However, they are shifted due to the formation of the hydrogen bond. The quinoid and benzenoid rings of C=C stretching vibration in PANI are responsible for the main distinctive peaks that show at 1587 and 1499 cm^–1^.^48^ The peaks at 3595, 2992, and 1324 cm^–1^ for PANI/MXene arise from hydrogen bond formation between the N–H, C–H, and C–N stretching vibrations of PANI at 3381, 3040, and 1308 cm^–1^ and the −OH, C–O, and O–H stretching vibrations of MXene at 3426, 2925, and 1381 cm^–1^.^49,50^ The FTIR peak shift indicates formation of the hydrogen bond between N–H, C–H, and C–N of PANI and −OH and −O of MXene (Figure 1a), leading to enhanced attraction between the PANI and MXene layers and possibly facilitating ion transport through local electric polarization.^51^ The ATR-FTIR test on the PANI/MXene thin film in the 1st and 50th cycles (Figure S13b), and the absorption peaks are almost unchanged, indicating good stabilization of the thin film.

The Raman peak shift also provides clear evidence for the hydrogen bond formation (Figures 1g and S2h,i. The Raman spectrum of the PANI/MXene thin film exhibits shifted characteristic peaks of PANI, i.e., C=C stretching of the quinonoid ring at 1570 cm^–1^, C–N stretching vibration at 1331 cm^–1^, and C–H in-plane bending at 1094 cm^–1^.^52−54^ The MXene’s shifted characteristic peaks are situated at 243, 404, and 559 cm^–1^, corresponding to in-plane vibrations of titanium, carbon, and surface groups, vibrations of Ti_3_C_2_F_2_, and vibrations of Ti_3_C_2_(OH)_2_.^55,56^Figure S3 displays the cross-sectional SEM image of the PANI/MXene thin film after 1000 CA cycles; MXene remains stable between PANI, with no aggreagation observed. It is further demonstrated that the sandwich structure protects MXene from direct exposure to air, thereby reducing its contact with molecular oxygen. Additionally, due to the flexibility of PANI, the MXene sandwiched between the PANI will not restack or aggregation.

**Figure 2:**
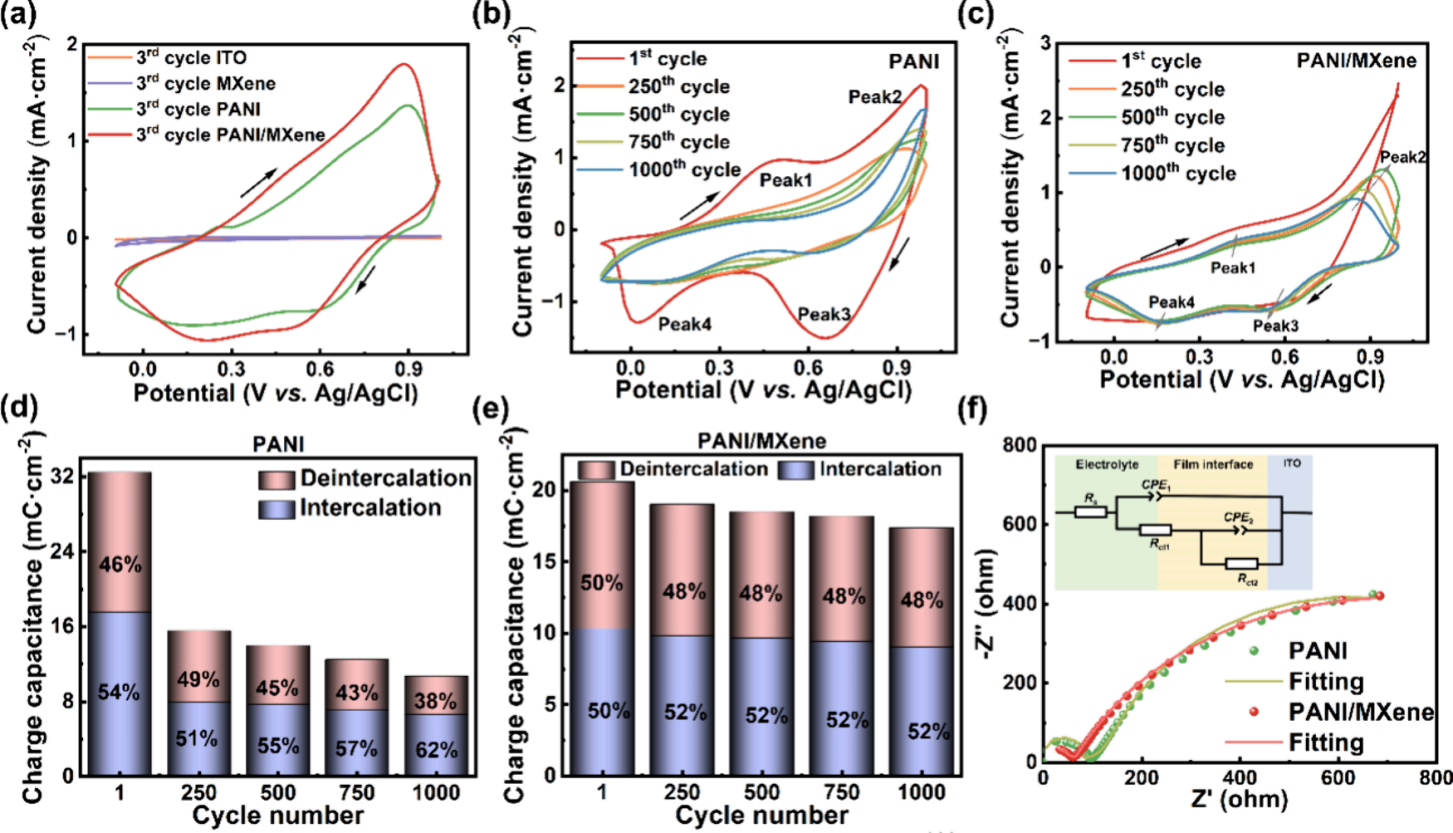
Electrochemical properties of thin films in the electrolyte of 0.01 M HCl/TsOH. (a) CV curves of various thin films. CV curves of (b) PANI and (c) PANI/MXene for the indicated cycles. Charge capacitance of (d) PANI and (e) PANI/MXene calculated from data in parts b and c. (f) Nyquist plots of the PANI and PANI/MXene thin films before cycling (the inset shows the circuit model).

Figure 2a shows the CV curves of the ITO, PANI, MXene, and PANI/MXene thin films at a scan rate of 50 mV·s^–1^. The current densities of the ITO layer and MXene thin film are 1.2 × 10^–5^ and 1.8 × 10^–2^ mA·cm^–2^, respectively, notably lower than those of the PANI and PANI/MXene thin films (1.4 and 1.8 mA·cm^–2^, respectively). This suggests that the increased surface area after the combination of MXene is the main factor contributing tothe PANI/MXene thin film’s charge capacitance, as indicated by the BET data mentioned above. As can be seen from the PANI and PANI/MXene thin film cycling CV curves (Figure 2b,c), there are two redox peaks in both figures. Peak 1 of the oxidation peak of the PANI/MXene thin film appears at a potential of 0.4 V and peak 2 appears at a potential of 0.9 V, which correspond to the oxidation of the thin film from the fully reduced state to the semioxidized state and from the semioxidized state to the fully oxidized state, respectively. The reduction peaks of peaks 3 and 4 appear at the potentials of 0.6 and 0.15 V, respectively. They correspond to the reduction of the thin film from the fully oxidized state to the semioxidized state and from the semioxidized state to the fully oxidized state. There is a clear presence of the four peaks in the PANI thin film in the first curve, but the peaks of the redox peaks gradually disappear after many curves of cycling, which indicates worse stability of the PANI thin film. The PANI thin film exhibits poor stability.

During the 1000th CV cycle, the PANI thin film suffers severe degradation with continuously reduced charge capacitance (Figure 2b). The proportion of the deintercalated charge is rapidly decreasing (Figure 2d), implying excessive intercalation of charge, leading to persistent expansion and damage of the chain skeleton of PANI to affect the cycling stability and energy storage performance. In comparison, the PANI/MXene thin film is much more durable (Figure 2c), and the ratio of the intercalated and deintercalated charge is close to 1 (Figure 2e), demonstrating superior reversibility. The spike observed in the first CV cycle is attributed to the significant change in the MXene layer spacing (Figure S4).^57^

EIS was further performed to investigate the charge transfer behavior. The Nyquist plots for the PANI and PANI/MXene thin films before cycling display two semicircles, and the impedance data align well with the circuit model shown in Figure 2f. In this model, CPE_1_ and CPE_2_ represent the double-layer capacitances, *R*_s_ denotes the resistance from the electrolyte, *R*_ct1_ corresponds to the charge transfer resistance at the electrolyte–film interface, and *R*_ct2_ represents the charge transfer resistance within the film. The PANI/MXene thin film demonstrates rapid charge transfer, as is evident from its lower *R*_ct1_ and *R*_ct2_ values compared to the PANI thin film (Table S1). This accelerated charge transfer in the PANI/MXene thin film contributes to enhanced electrochromic and energy storage performance. The third cycle performed similarly to the first cycle (Figure S5a and Table S1). During the 1000th cycle, the shape of the impedance response shows a semicircle with a tail on its right side (Figure S5b). The larger slope of the PANI/MXene thin film in the low-frequency region also indicates the faster ion diffusion and charge transfer rate.

**Figure 3:**
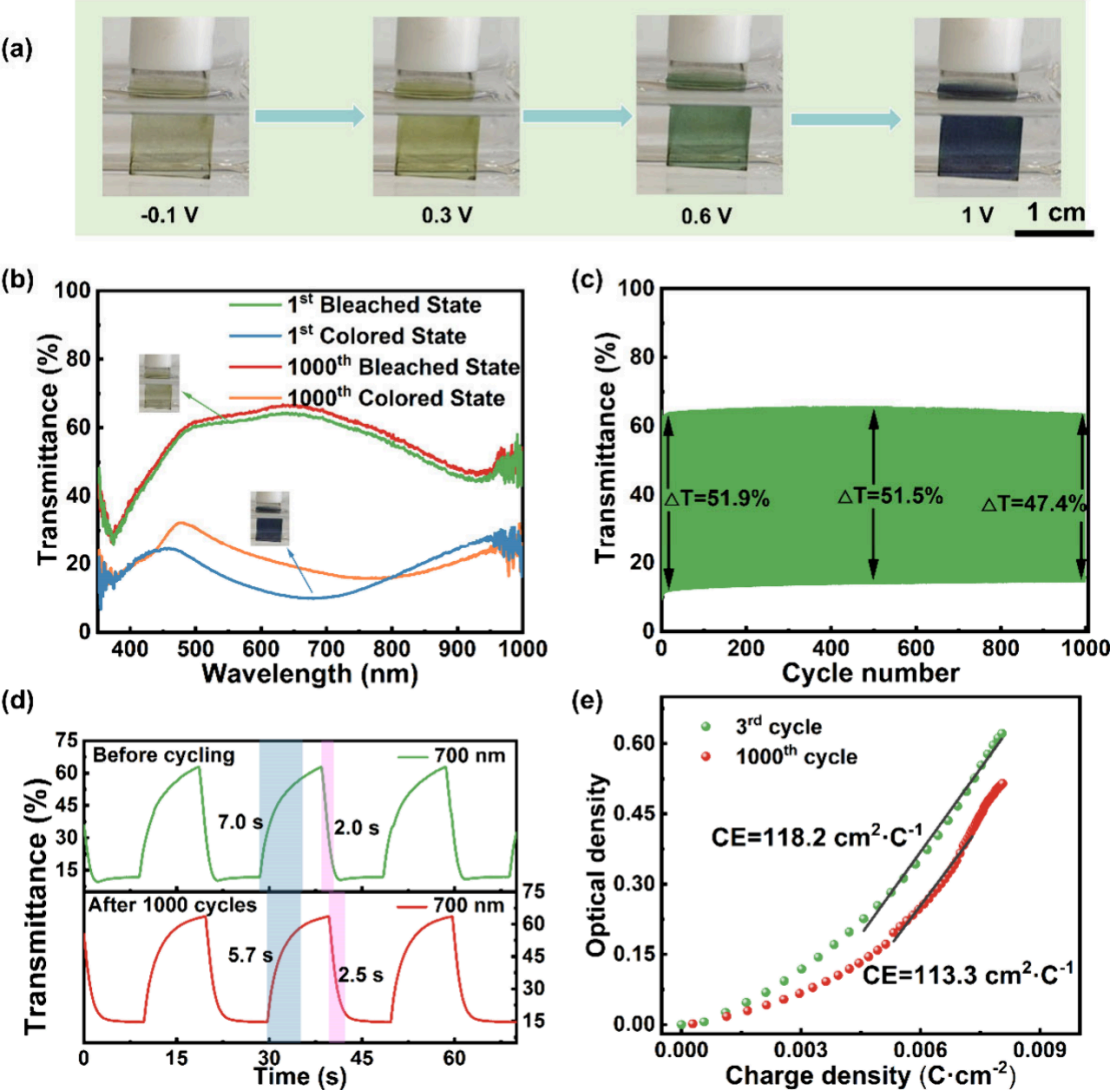
Electrochromic properties of the PANI/MXene thin film in the electrolyte of 0.01 M HCl/TsOH. (a) Photographs of the film for the indicated potentials. (b) Transmittance spectra recorded. (c) Optical durability during 1000 CV cycles at a wavelength of 700 nm. (d) Switching time. (e) Coloration efficiency.

The PANI/MXene thin film at the given potentials during the electrochromic process is photographed in Figure 3a. The multicolor switch was from PANI (Figure S6a), as illustrated in eqs 9–11. However, the PANI/MXene thin film has a wider tunable spectral range, with a reversible light-yellow-to-dark-blue color change (Figure S7 and Table S2). The electrochromic properties of essential importance include optical transmittance modulation, optical durability, switching time, and coloration efficiency. For the optical transmittance modulation, the initial value of the PANI/MXene thin film is 51.9% (*T*_b_ = 62.1% and *T*_c_ = 10.2%), and it retains 47.4% after 1000 CV cycles (Figure 3b,c). In contrast, optical modulation of the PANI thin film presents a severe degradation and is decreased by 65.9% (Figure S6b,c). Upon comparison of the photographs of the two degraded thin films after 1000 CV cycles, the PANI/MXene thin film color switch is maintained, whereas the PANI thin film color switch is limited to light and dark blue (Figures S8 and S9 and Table S3).

The switching time and coloration efficiency are determined by CA cycling by using an applied potential of −0.1 and 1.0 V for 10 s each. The switching time reflects the switching rate of electrochromic materials and is the time required to achieve a 90% transition between the bleached and colored states. The PANI/MXene thin film coloring and bleaching times are 2.0 and 7.0 s before cycling and 2.5 and 5.7 s after 1000 CA cycles (Figure 3d), faster than the color switch of the PANI thin film (Figure S6d). The coloration efficiency of the PANI/MXene thin film is 118.2 cm^2^·C^–1^ at the beginning and 113.3 cm^2^·C^–1^ after cycling (Figure 3e), exhibiting persistent high coloration efficiency compared with the PANI thin film (Figure S6e).

**Figure 4:**
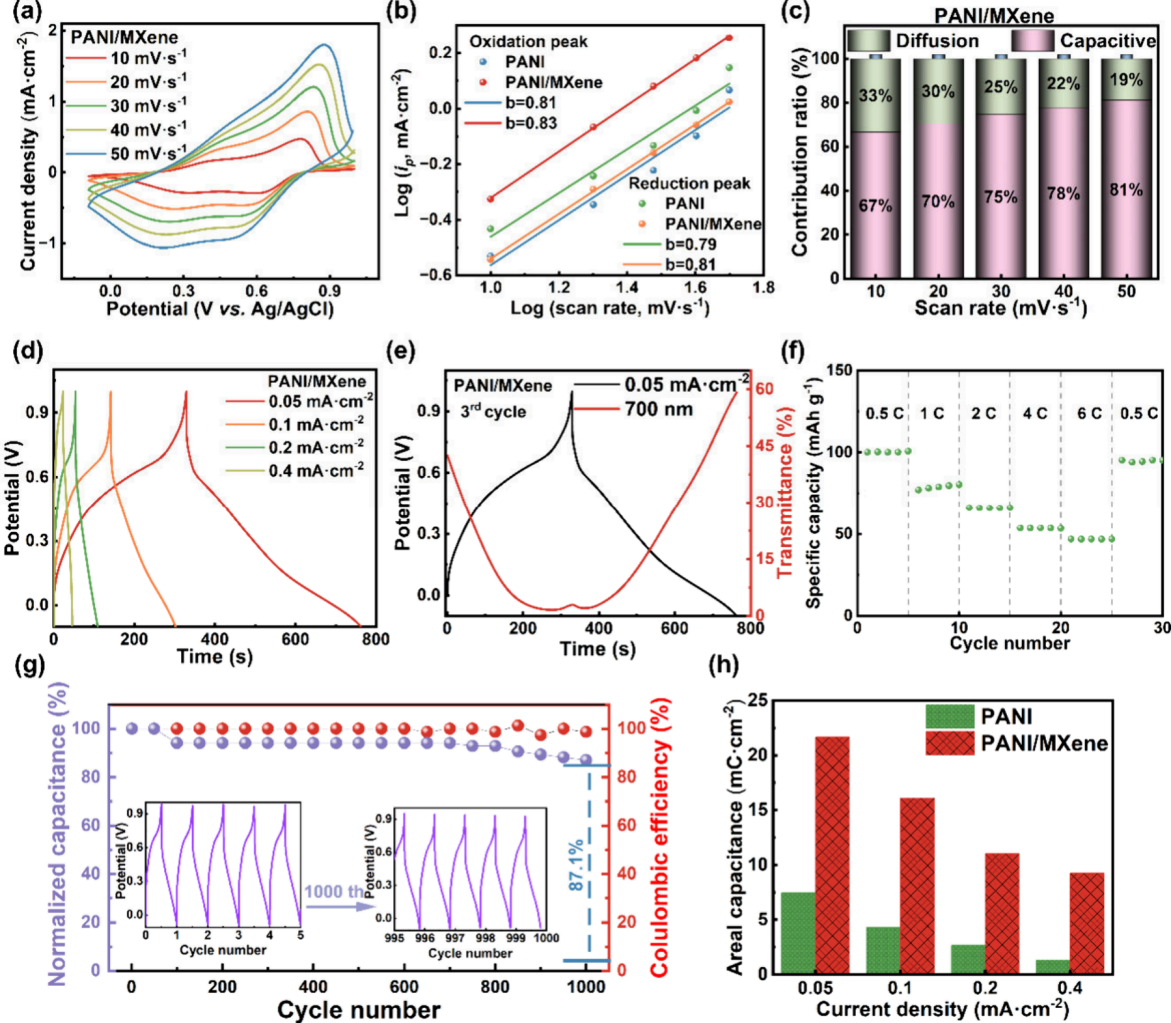
Energy storage characteristics of the PANI/MXene thin film. (a) CV curves at different scan rates. (b) Logarithmic relationship between the peak current density (*i*_p_) of oxidation/reduction and scan rate. (c) Contribution of capacitance (purple area) to the overall current at different scan rates. (d) GCD curves at different current densities of −0.1 to 1 V. (e) 3rd GCD cycle curve and corresponding in situ transmittance. (f) Rate performance. (g) Electrochemical durability and Coulombic efficiency over 1000 GCD cycles at 0.4 mA·cm^–2^. The inset represents the initial and last five cycles. (h) Areal capacitance versus current density.

CV and GCD tests were performed on PANI and PANI/MXene thin films to investigate their energy storage performance. Both thin films have obvious redox peaks during the scan process within the potential range of −0.1 to 1 V; the CV curves at different scan rates are of similar shape but the redox peaks shift a little, exhibiting a quasi-reversible characteristic (Figures 4a and S10a). The charge storage behavior of thin films is then identified using the power-law rule (eq 3, 4), as shown in Figure 4b. The thin film’s two *b* values, which range from 0.5 to 1, reveal that the charge storage behavior is mixed and involves both diffusion-controlled and capacitance-controlled kinetic mechanisms. The capacitive control dominates as *b* approaches 1, which is in agreement with the observations in Figures 4c and S11. It should be mentioned that a perfect linear connection (*R*_2_ = 0.99) is obtained between the square root of the scan rate (*v*^1/2^) and the peak current density (*i*_p_), suggesting that a semi-infinite diffusion process controls the electrochemical process (Figure S12). However, the high diffusion coefficients, on the order of 10^–8^, observed in both the PANI and PANI/MXene thin films facilitate rapid diffusion, resembling charge transfer behavior. This effect is particularly pronounced in PANI/MXene, which exhibits a larger *b* value and higher diffusion coefficients, contributing to faster reaction kinetics and excellent cycling stability.

Figures 4d and S10b display the GCD curves for PANI/MXene and PANI thin films. The longer discharge time observed in the PANI/MXene thin film indicates a larger specific capacitance. The optical transmittance modulation curve at 700 nm during GCD cycling illustrates the reversible color switch during the charging/discharging processes (Figures 4e and S10c). The PANI/MXene thin film exhibits excellent rate performance at the current rate from 0.5 to 6 C (Figure 4f).

Figure 4g displays the capacity retention and Coulombic efficiency of the PANI/MXene thin film during GCD cycling. After 1000 GCD cycles, it retains 87.1% of its initial capacitance and an impressive 99.0% Coulombic efficiency, demonstrating significantly greater durability compared to the PANI thin film (Figure S10d). Furthermore, Figure 4h highlights a considerable increase in areal capacitance for the PANI/MXene thin film. Specifically, the PANI/MXene reveals an area capacitance of 21.6 mC·cm^–2^ at 0.05 mA·cm^–2^, markedly surpassing the 7.4 mC·cm^–2^ of the PANI. The incorporation of MXene led to an increased specific surface area and enhanced electrochemical activity, which contributed to the superior electrochromic and energy storage performance of the PANI/MXene thin film (Table S4).

**Figure 5:**
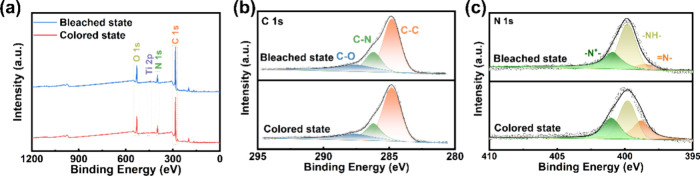
XPS spectra of PANI/MXene thin films in their colored and bleached states. (a) XPS survey spectra. High-resolution XPS spectra of (b) C 1s and (c) N 1s.

XPS measurement was carried out to analyze the redox reaction of the PANI/MXene thin film in the electrochromic and energy storage process (Figure 5). Figure 5a shows the survey spectra of the PANI/MXene thin film in the bleached and colored states. Characteristic peaks of C, O, N, and Ti can be observed, and the weak peaks of Ti are because MXene is below PANI.^58^ MXene and PANI are not fully mixed but touch at their interface. Because XPS can only penetrate several nanometers, the Ti 2p peak that we measured is very weak (Figure S13a). Figure 5b shows the high-resolution C 1s XPS spectrum with peaks corresponding to various carbon bonds: C–C (284.8 eV), C–N (286.2 eV), and C–O (287.6 eV).^59^ During the redox process, the atomic fractions of C–C, C–N, and C–O do not change much (Table S4). High-resolution XPS spectra of N 1s are displayed in Figure 5c. The quinone ring (=N−) is represented by the peak at 398.8 eV in the colored state, while the peaks of aniline (−NH−) and protonated–N^+^– are located at 399.8 and 400.6 eV, respectively.^41^ During the coloration process, anions (Cl^–^ and TsO^–^) are embedded in the thin film to adsorb nitrogen atoms. This leads to decreased atomic fractions of −NH– and −N^+^– and the increased atomic fraction of =N–. In contrast, the bleaching process undergoes the opposite change (Table S5). Therefore, the electrochromic and energy storage process of the PANI/MXene thin film mainly involves the valence state change of N. It should be mentioned that the coloration process is also the charging process, while the reversible bleaching process corresponds to the discharging process.^60,61^
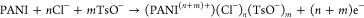
9
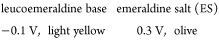

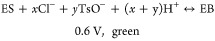
10
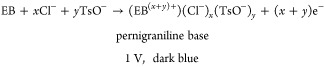
11

**Figure 6:**
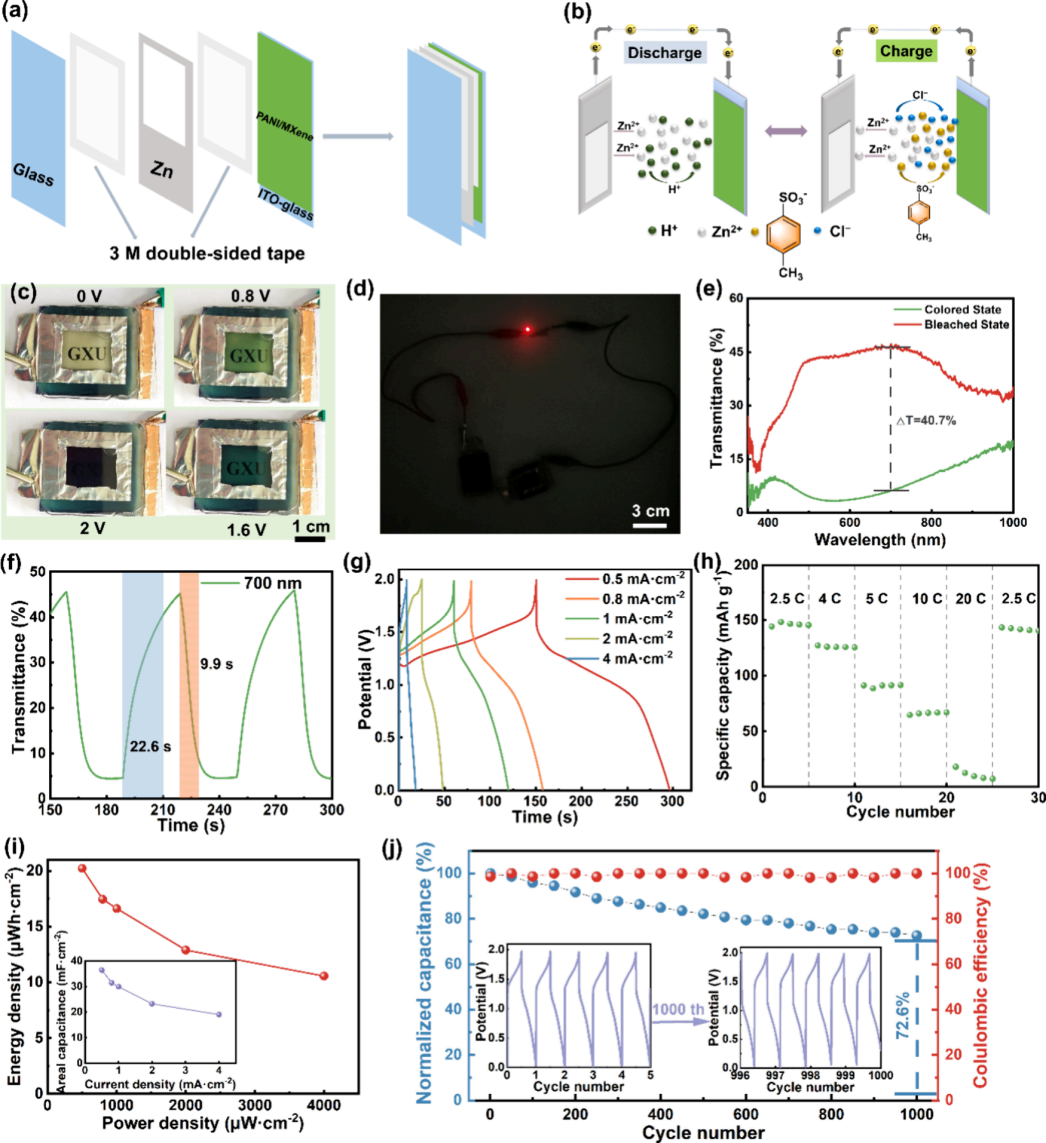
Electrochromic energy storage capability of the PANI/MXene device for Zn^2+^ storage. Schematic illustration of (a) the device assembly and (b) the electrochromic energy storage mechanism. (c) Photographs for the indicated voltages. (d) Red LED powered by two devices in a dark environment. (e) Optical transmittance modulation. (f) Switching time. (g) GCD curves at different current densities (potential range: 0–2 V). (h) Rate performance. (i) Ragone plot. (j) Electrochemical durability and Coulombic efficiency during 1000 GCD cycles at 4 mA·cm^–2^. The inset represents the initial and last five cycles.

The PANI/MXene device is also fabricated (Figure 6a) to examine the energy storage and electrochromic performance. The coloration and charging/discharging mechanism of the device are depicted in Figure 6b. The electrochromic and capacitive behavior of PANI/MXene primarily depends on the reversible redox reaction of PANI, as seen by the CV curves with significant differences in the area (Figure 2a).

The following are the relevant reaction equations:Bleaching/discharging process:
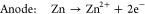
12
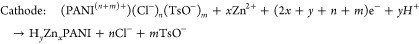
13Coloration/charging process:
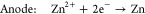
14
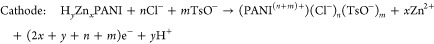
15

During the reversible bleaching/discharging and coloration/charging processes, the device’s color switches between light yellow and dark blue (Figure 6c) and can light up a red LED (Figure 6d). Quantitative color specifications are shown in the chromaticity diagram of Figure S14, and Table S6 displays the information corresponding to the chromaticity coordinates *x* and *y*.

By applying pulsed potentials of 0 and 2.0 V for 30 s each, CA cycling can evaluate the electrochromic property of the device. It exhibits an optical transmittance modulation of 40.7% (Figure 6e) and coloring/bleaching times of 9.9 s/22.6 s, respectively (Figure 6f). The coloration efficiency at 700 nm is 75.0 cm^2^·C^–1^ (Figure S15a).

The device’s GCD curves are displayed in Figure 6g across different current densities within a voltage range of 0–2 V. The computed areal capacitances are 72.8, 62.8, 59.8, 46.4, and 41.4 mC·cm^–2^ at 0.5, 0.8, 1.0, 2.0, and 4.0 mA·cm^–2^, respectively. Figure 6h illustrates the device’s exceptional rate performance across 2.5–20 C. The Ragone plot in Figure 6i exhibits the device’s energy density of 20.2 μWh·cm^–2^ and power density of 500.7 μW·cm^–2^, maintaining an energy density of 10.6 μWh·cm^–2^ even at a high-power density of 4000.0 μW·cm^–2^. The device also showed remarkable durability at 4 mA·cm^–2^ (transmittance modulation retention of 91.5%) and transmittance modulation (∼30%) with capacitance retention of 72.6%, and the Coulombic efficiency remains above 98% during 1000 GCD cycles (Figure 6j and S15b,c). The device demonstrates superior durability and coloration efficiency compared to previously reported works, as shown in Table S7.

## 4 Conclusions

In summary, the PANI/MXene composite thin film has been successfully prepared to overcome the limitation of specific surface area and the excess intercalation of charge, to achieve excellent electrochromic and energy storage performance. Hydrogen bonds formed between N–H, C–H, C–N of PANI and −OH and −O surface functional terminations of MXene enhance the interface interaction between the PANI and MXene layers. The PANI/MXene thin film features large optical transmittance modulation (∼50% during 1000 CA cycles), remarkable coloration efficiency (700 nm, 118.2 cm^2^·C^–1^), large areal capacitance (0.05 mA·cm^–2^ and 21.6 mC·cm^–2^), excellent optical durability and electrochemical durability (87.1% capacitance retention after 1000 cycles). Moreover, the PANI/MXene electrochromic energy storage device is also fabricated and investigated. It exhibits superior electrochromic performance (optical transmittance modulation, 40.7%; coloration efficiency, 75.0 cm^2^·C^–1^), Zn^2+^-storage performance (areal capacitance, 72.8 mC·cm^–2^; energy density, 20.2 μWh·cm^–2^; power density, 500.7 μW·cm^–2^; 72.6% capacitance retention after 1000 cycles). Our work provides new insights into the design of electrochromic and energy storage materials and devices, with findings that are also applicable to other types of ionic devices.

## Supporting Information Available

Supportive data concerning FTIR, BET, Raman, SEM, XPS, and SEM characterization, electrochromic properties, and energy storage characteristics of the PANI thin film (PDF)
