# Supplementary material for: Layer-by-Layer-Assembled Polyaniline/MXene Thin Film and Device for Improved Electrochromic and Energy Storage Capabilities
Source: ACS Appl Polym Mater. 2024 Oct 9;6(20):12492–502. doi: 10.1021/acsapm.4c01774 (PMC11519834; doi:10.1021/acsapm.4c01774)
Supplement: Supplementary file 1 — ap4c01774_si_001.pdf [file ap4c01774_si_001.pdf]

Supporting Information

Layer–by–Layer Assembled Polyaniline/MXene Thin Film and Device for Improved Electrochromic and Energy Storage Capabilities

Dejuan Lu [a], *Jian Li* [a], *Dashui Zhang* [a], *Lina Li* [a], *Zhangfa Tong* [a], *Hongbing Ji* [a], *Junxin Wang* [*b], *Caixia Chi* [*c], *and Hui-Ying Qu* [*a]

[a] Guangxi Key Laboratory of Petrochemical Resource Processing and Process Intensification Technology, School of Chemistry and Chemical Engineering, Guangxi University, Nanning 530004, China

[b] Department of Materials Science and Metallurgy, University of Cambridge, 27 Charles Babbage Road, Cambridge CB3 0FS, United Kingdom

[c] Heilongjiang Province Key Laboratory of Environmental Catalysis and Energy Storage Materials, Food and Pharmaceutical Engineering College, Suihua University, Suihua 152061, China

*Corresponding authors. E-mails: wangjunxin2010@gmail.com (J. Wang), 26078396@qq.com (C. Chi), huiying.qu@gxu.edu.cn (H-Y. Qu).

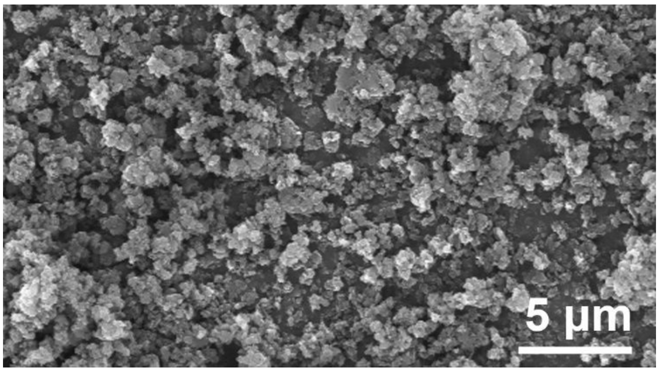

Figure S1. A surface SEM image of the PANI powder.

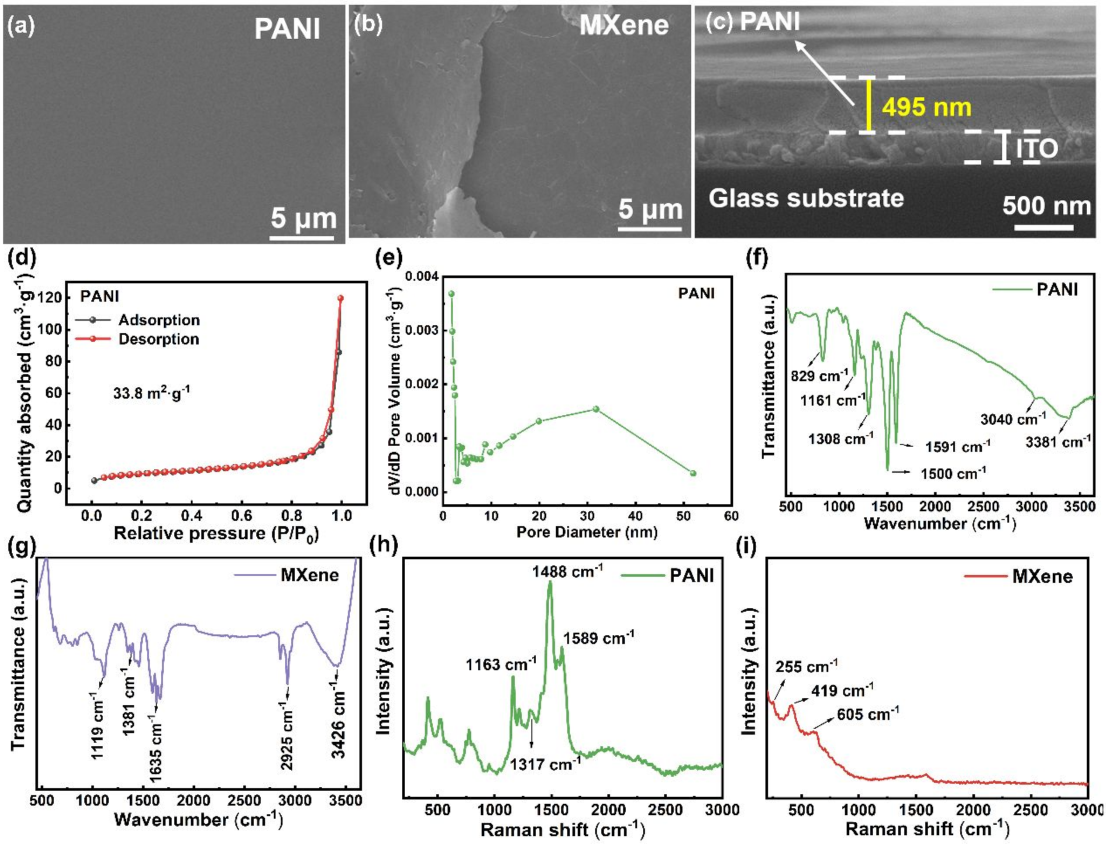

Figure S2. Surface SEM images of the (a) PANI and (b) MXene thin films. (c) Cross-sectional SEM image of the PANI thin film. (d) N_2 adsorption/desorption isotherms of the PANI powder. (e) Pore size distribution of the PANI powder. FTIR spectra of the (f) PANI powder and (g) MXene powder. Raman spectra of the (h) PANI thin film and (i) MXene thin film.

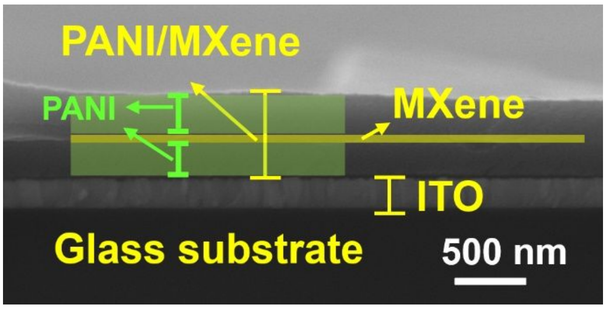

Figure S3. Cross-sectional SEM image of the PANI/MXene thin film after 1000 CA cycles.

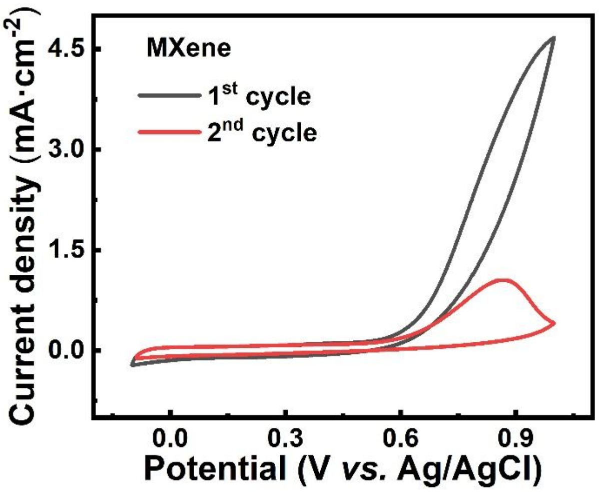

Figure S4. CV curves for the MXene thin film during the first and second cycles.

Table S1. Comparison of EIS data.

	Rs/ohm (PANI, PANI/MXene)	R_{ct1}/ohm (PANI, PANI/MXene)	R_{ct2}/ohm (PANI, PANI/MXene)
1st	94.6, 58.9	304.2, 131.6	1266, 1253
3rd	60.4, 51.6	55.8, 48.6	95.8, 65.3

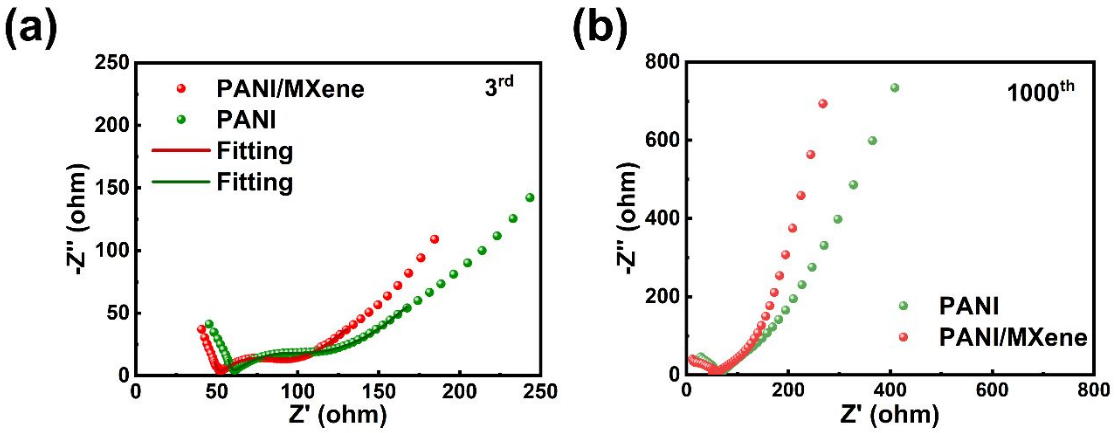

Figure S5. Nyquist plots of the PANI and PANI/MXene thin films in the (a) 3rd and (b) 1000th cycles.

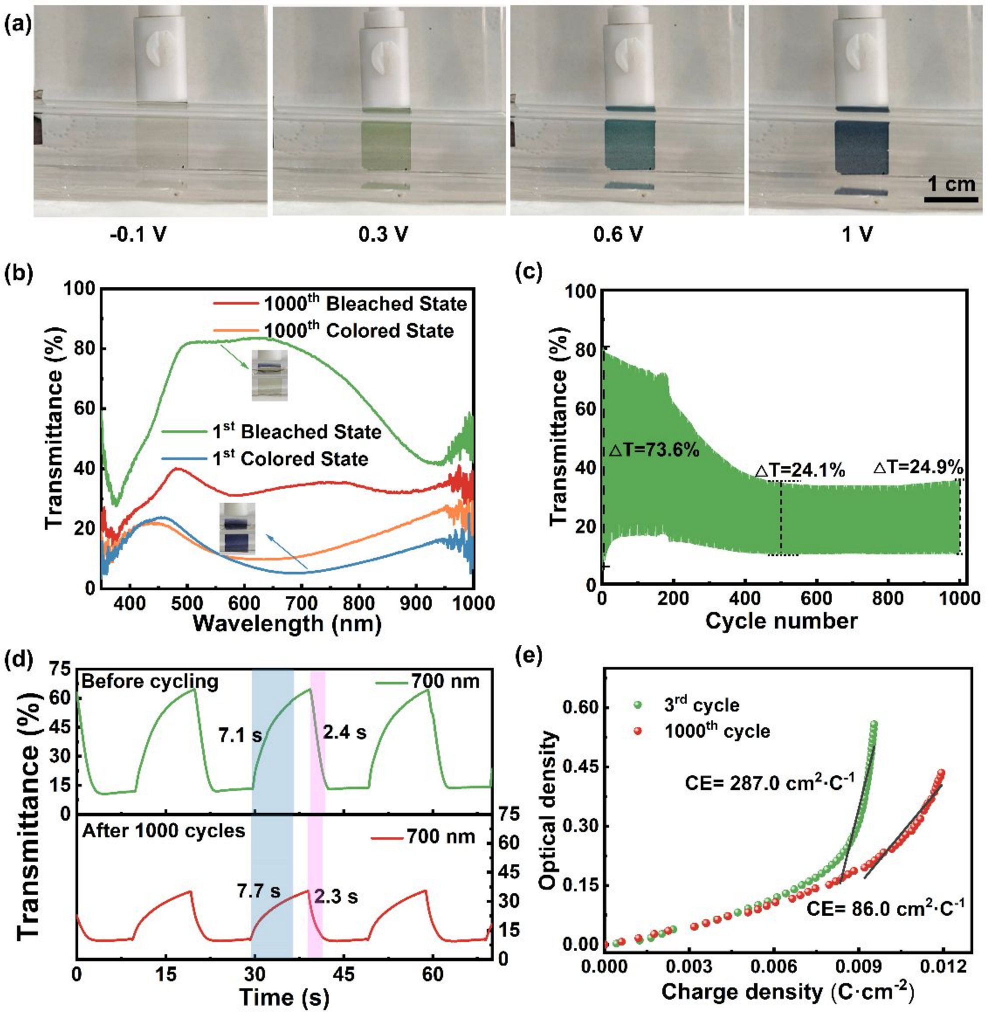

Figure S6. Electrochromic performance of the PANI thin film in the electrolyte of 0.01 M HCl/TsOH. (a) Photographs of the film at specified potentials. (b) Transmittance spectra recorded during 1000 CV cycles. (c) Optical durability measured during 1000 CV cycles at the wavelength of 700 nm. (d) Switching time. (e) Coloration efficiency.

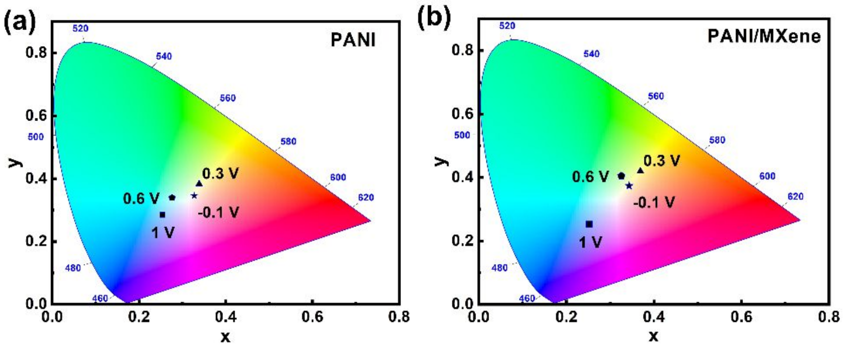

Figure S7. CIE 1931 chromaticity diagram with (x, y) coordinates for the (a) PANI and (b)

PANI/MXene thin films.

Table S2. Chromaticity coordinates (*x*, *y*) derived from Figure S7 for the PANI and PANI/MXene thin films, respectively.

Potential	PANI (*x*, *y*)	PANI/MXene (*x*, *y*)
–0.1 V	(0.32747,0.34552)	(0.34339,0.37379)
0.3 V	(0.33870,0.38288)	(0.36909,0.41980)
0.6 V	(0.27628,0.3393)	(0.32569,0.40512)
1 V	(0.25422,0.28507)	(0.25254,0.25217)

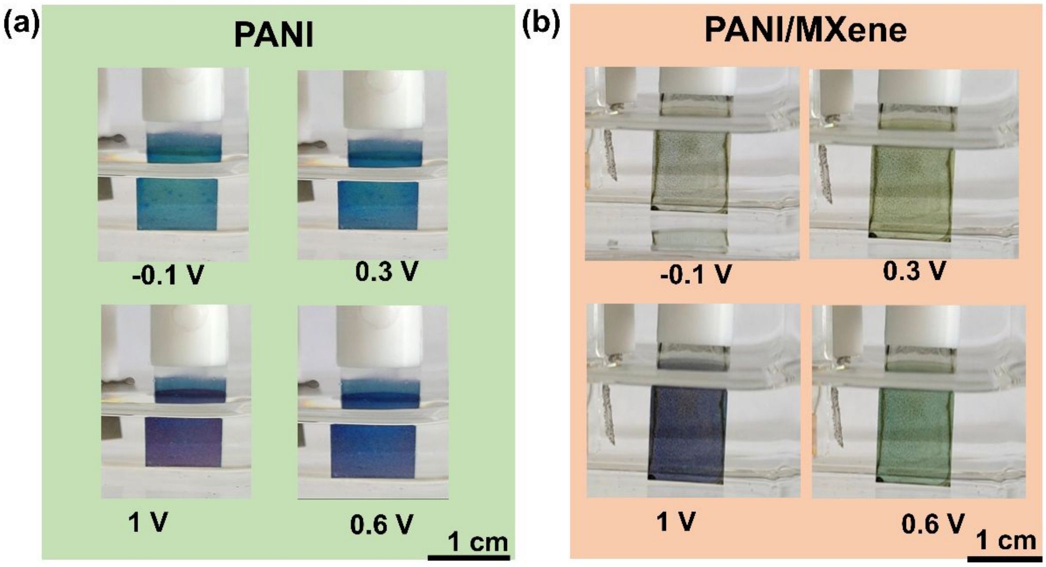

Figure S8. Photographs of the thin films after 1000 CA cycles. (a) PANI, (b) PANI/MXene.

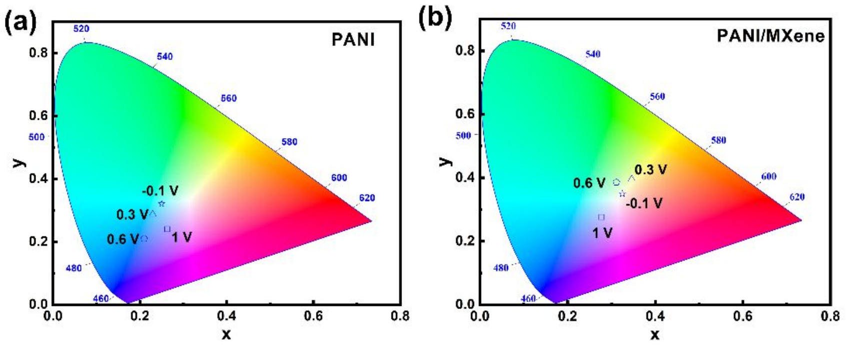

Figure S9. CIE 1931 chromaticity diagram with (*x*, *y*) coordinates for the (a) PANI and (b) PANI/MXene thin films after 1000 CA cycles. (*x*, *y*) data are calculated from RGB values extracted

by Photoshop from the corresponding photos.

Table S3. Chromaticity coordinates (*x*, *y*) derived from Figure S9 for the PANI, PANI/MXene thin films, respectively.

Potential	PANI (*x*, *y*)	PANI/MXene (*x*, *y*)
–0.1 V	(0.25020, 0.32084)	(0.32502, 0.34935)
0.3 V	(0.22948, 0.28792)	(0.34615, 0.39563)
0.6 V	(0.20981, 0.21000)	(0.31128, 0.38573)
1 V	(0.26289, 0.24018)	(0.27748, 0.27563)

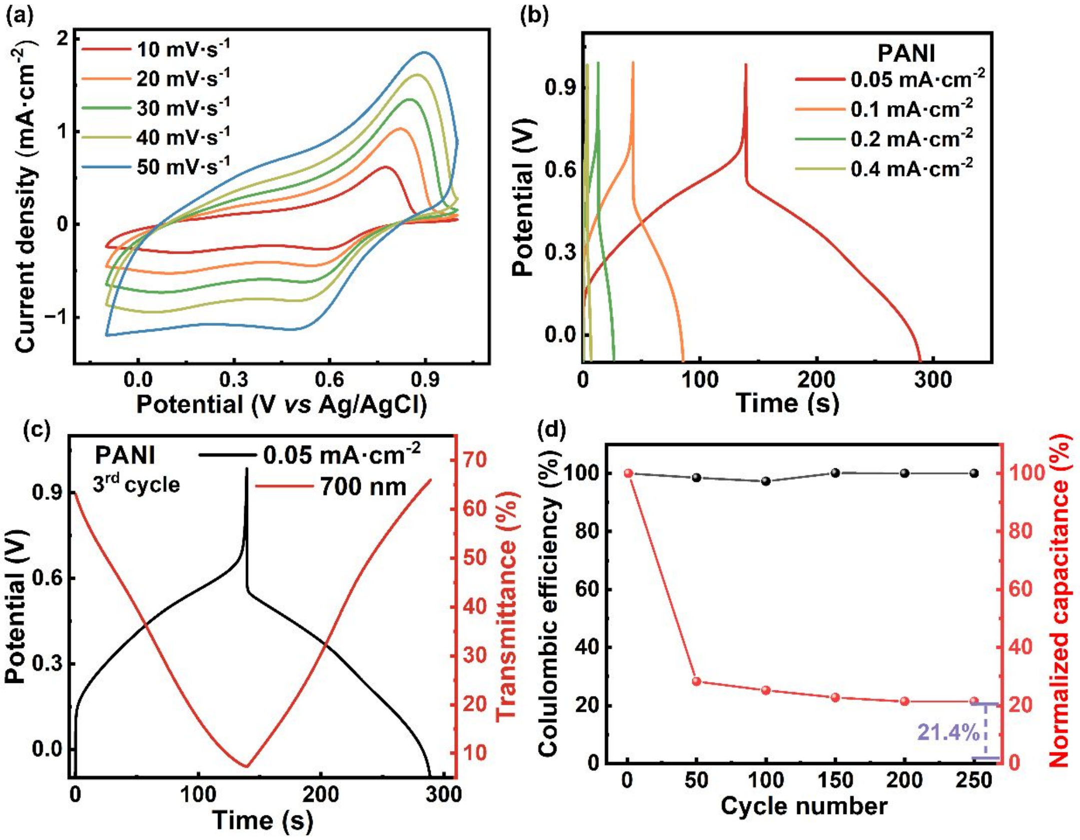

Figure S10. Electrochromic energy storage capabilities of the PANI thin film. (a) CV curves at various scan rates. (b) GCD curves at various current densities in the potential range of –0.1 V to 1 V. (c) The 3rd GCD cycle curve and its corresponding in-situ transmittance at 700 nm at a current density of 0.05 mA·cm^{-2}. (d) Electrochemical stability and coulombic efficiency during 250 GCD

cycles at a current density of 0.4 mA·cm^{-2}.

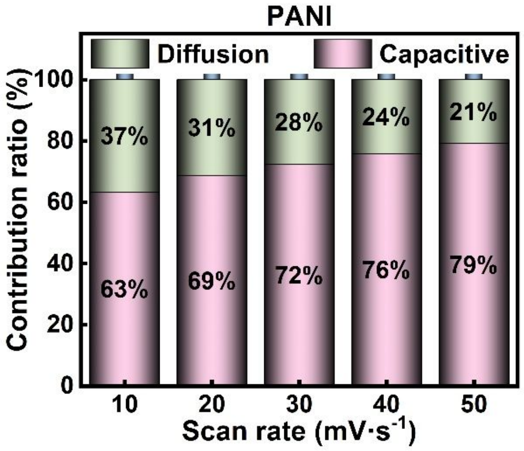

Figure S11. Contribution of capacitance (purple area) to the total current at various scan rates of the PANI thin film.

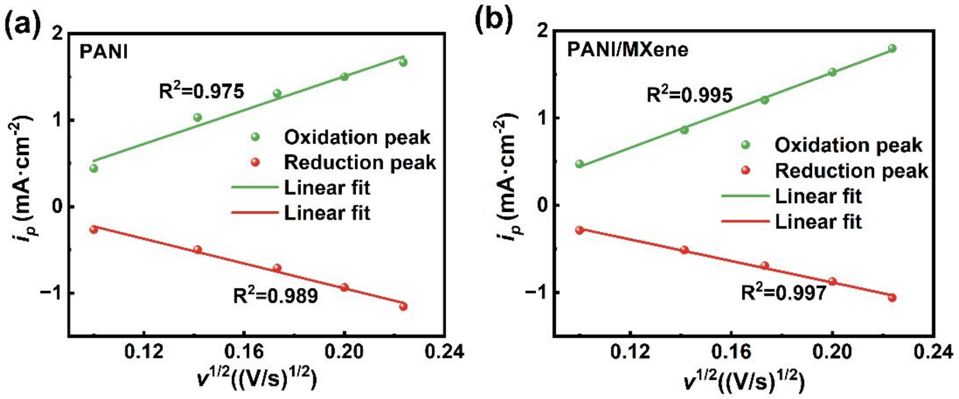

Figure S12. Redox peak current (i_p) versus $v^{1/2}$ to calculate the diffusion coefficients (a) of the PANI and (b) PANI/MXene thin films by the Randles-Sevcik equation:

$$i_p = 0.4463zFA\left(\frac{zF}{RT}\right)^{1/2} D^{1/2}cv^{1/2}$$

where i_p is the peak current, z is the number of electrons transferred in a unit reaction (for PANI, z is generally taken as 2), F is the Faraday constant, A is the effective surface area of the electrode, R is the ideal gas constant, T is the experimental temperature, D is the effective diffusion coefficient, c is the concentration of ions involved in the reaction and v is the potential scan rate. The oxidation and reduction diffusion coefficients for PANI are 1.69 E–8 cm^2·s^{-1}, 1.64 E–8 cm^2·s$^-$

[1]. The oxidation and reduction diffusion coefficients for PANI/MXene are 1.71 E–8 $cm^2·s^{-1}$, 1.72 E–8 $cm^2·s^{-1}$.

Table S4. Comparison of the bi-functional PANI-based films.

Materials	Transmittance modulation	Coloration efficiency	Optical durability	Areal capacitance	Reference
PANI/WO_3	35.3%@ 633 nm	98.4 $cm^2·C^{-1}$	NA	12.0 $mF·cm^{-2}$@ 0.008 $mA·cm^{-2}$	[1]
TiO_2/PANI	76.9%@ 600 nm	78 $cm^2·C^{-1}$	89.4%, 100 cycles	7.0 $mF·cm^{-2}$@ 0.05 $mA·cm^{-2}$	[2]
GO/PANI	25%@ 700 nm	41.02 $cm^2·C^{-1}$	66%, 500 cycles	75 $mF·cm^{-2}$@ 0.075 $mA·cm^{-2}$	[3]
WO_3/PANI<br>WO_3/graphene/PANI	9.04%@ 633 nm<br>4.52%@ 633 nm	40.42 $cm^2·C^{-1}$<br>67.47 $cm^2·C^{-1}$	NA	4.312 $mF·cm^{-2}$@ 0.08 $mA·cm^{-2}$<br>11.267 $mF·cm^{-2}$@ 0.08 $mA·cm^{-2}$	[4]
PANI@MXene	90%@1079 nm	210.48 $cm^2·C^{-1}$	94.3%, 1000 cycles	NA	[5]
PANI/MXene	55%@700 nm	NA	87.3%, 500 cycles	NA	[6]
**PANI/MXene**	**51.9%@ 700 nm**	**118.2 $cm^2·C^{-1}$**	**91.3%, 1000 cycles**	**19.7 $mF·cm^{-2}$ (21.6 $mC·cm^{-2}$) @ 0.05 $mA·cm^{-2}$**	**This work**

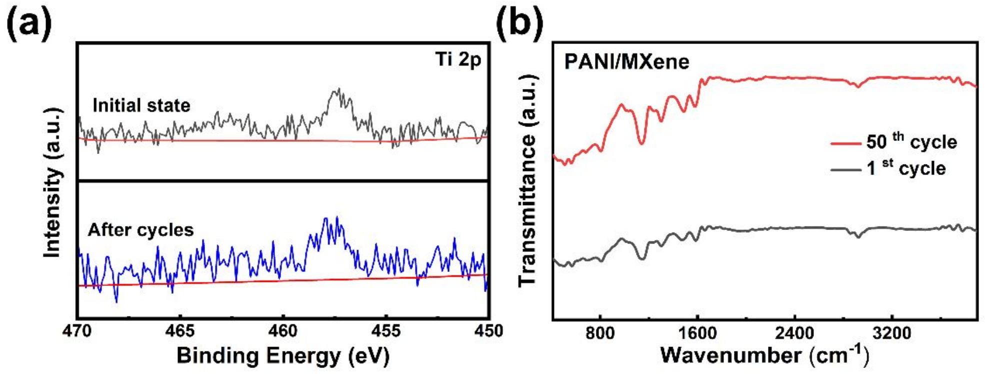

Figure S13. (a) High-resolution XPS spectra of Ti 2p of the PANI/MXene thin film. (b) ATR-FTIR spectra of the PANI/MXene thin film in the 1st and 50th cycle.

Table S5. Atomic fractions of each orbital of PANI/MXene thin films calculated from XPS data (in at.%)

Atomic orbita			Colored state	Bleached state
**C 1s**	C–C/C=C		66.1	62.4
	C–N		20.9	20.9
	C–O		13.0	16.6
**N 1s**	=N–		28.8	14.2
	–NH–		44.1	57.2
	$–N^{+}–$		27.1	28.6

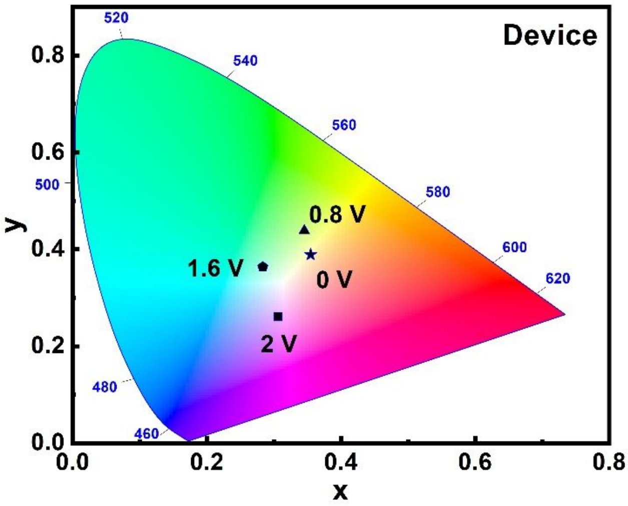

Figure S14. CIE 1931 chromaticity diagram with (*x*, *y*) coordinates for the PANI/MXene electrochromic energy storage device. (*x*, *y*) data are calculated from RGB values extracted by Photoshop from the corresponding photos.

Table S6. Chromaticity coordinates (*x*, *y*) derived from Figure S14 for the PANI/MXene electrochromic energy storage device.

Potential	0 V	0.8 V	1.6 V	2 V
(*x*, *y*)	(0.35492,0.38885)	(0.34548,0.43828)	(0.28358,0.36342)	(0.30654,0.26128)

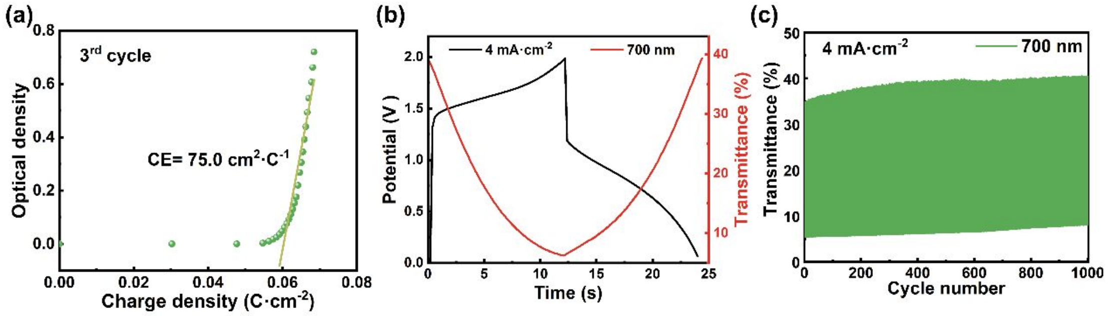

Figure S15. Electrochromic performance of the PANI/MXene device. (a) Coloration efficiency. (b) GCD curve at a current density of 4 mA cm^{-2} and its corresponding in-situ transmittance modulation at 700 nm. (c) Durability during 1000 GCD cycles at a current density of 4.0 mA cm^{-2}.

Table S7. Comparison of the bi-functional PANI-based devices.

Materials	Transmittance modulation	Coloration efficiency	Areal capacitance	Capacitance retention	Energy density /Power density	Reference
$W_{18}O_{49}$/PANI	25.8%@632 nm	45.7 $cm^2 C^{-1}$	52.96 $mAh \cdot g^{-1}$@ 0.5 $A \cdot g^{-1}$	-	NA	[7]
PANI/GO	60.6%@633 nm	59.3 $cm^2 C^{-1}$	19.2 $mF \cdot cm^{-2}$@ 0.008 $mA \cdot cm^{-2}$	53.1%, 1000 cycles	~2.25 $\mu W \cdot h \cdot cm^{-2}$ /~10 $\mu W \cdot cm^{-2}$	[8]
**PANI/MXene**	**40.7%@700 nm**	**75.0 $cm^2 C^{-1}$**	**36.4 $mF \cdot cm^{-2}$@ 0.5 $mA \cdot cm^{-2}$**	**72.6%, 1000 cycles**	**20.2 $\mu W \cdot h \cdot cm^{-2}$ /500.7 $\mu W \cdot cm^{-2}$**	**This work**

References

1. Wei, H.; Yan, X.; Wu, S.; Luo, Z.; Wei, S.; Guo, Z., Electropolymerized Polyaniline Stabilized Tungsten Oxide Nanocomposite Films: Electrochromic Behavior and Electrochemical Energy Storage. *The Journal of Physical Chemistry C* **2012,** *116* (47), 25052-25064.

2. Zhang, S.; Lei, P.; Fu, J.; Tong, X.; Wang, Z.; Cai, G., Solution-processable multicolor TiO_2/polyaniline nanocomposite for integrated bifunctional electrochromic energy storage device. *Applied Surface Science* **2023,** *607*.

3. Zhang, S.; Chen, S.; Cao, Y.; Yang, F.; Peng, H.; Yan, B.; Jiang, H.; Gu, Y.; Xiang, M., Polyaniline nanoparticle coated graphene oxide composite nanoflakes for bifunctional multicolor electrochromic and supercapacitor applications. *Journal of Materials Science: Materials in Electronics* **2019,** *30* (14), 13497-13508.

4. Lyu, H., Triple Layer Tungsten Trioxide, Graphene, and Polyaniline Composite Films for Combined Energy Storage and Electrochromic Applications. *Polymers* **2019,** *12* (1).

5. Liu, Y.; Yuan, J.; Wang, Y.; Zheng, R.; Liu, Q.; Shang, X.; Shao, J.; Wan, Z.; Luo, J.; Jia, C., Approach to Significantly Enhancing the Electrochromic

Performance of PANi by In Situ Electrodeposition of the PANi@MXene Composite Film. *ACS Applied Materials & Interfaces* **2023,** *15* (50), 58940-58954.
6. Lin, T.; Liu, W.; Yan, B.; Li, J.; Lin, Y.; Zhao, Y.; Shi, Z.; Chen, S., Self-Assembled Polyaniline/$Ti_3C_2T_x$ Nanocomposites for High-Performance Electrochromic Films. *Nanomaterials (Basel)* **2021,** *11* (11).
7. Chang, X.; Hu, R.; Sun, S.; Liu, J.; Lei, Y.; Liu, T.; Dong, L.; Yin, Y., Sunlight-charged electrochromic battery based on hybrid film of tungsten oxide and polyaniline. *Applied Surface Science* **2018,** *441*, 105-112.
8. Wei, H.; Zhu, J.; Wu, S.; Wei, S.; Guo, Z., Electrochromic polyaniline/graphite oxide nanocomposites with endured electrochemical energy storage. *Polymer* **2013,** *54* (7), 1820-1831.
